# Parallel-META 2.0: Enhanced Metagenomic Data Analysis with Functional Annotation, High Performance Computing and Advanced Visualization

**DOI:** 10.1371/journal.pone.0089323

**Published:** 2014-03-03

**Authors:** Xiaoquan Su, Weihua Pan, Baoxing Song, Jian Xu, Kang Ning

**Affiliations:** 1 Shandong Key Laboratory of Energy Genetics, CAS Key Laboratory of Biofuels and BioEnergy Genome Center, Computational Biology Group of Single Cell Center, Qingdao Institute of Bioenergy and Bioprocess Technology, Chinese Academy of Sciences. Qingdao, People's Republic of China; 2 School of Computer Science and Technology, University of Science and Technology of China, Hefei, Anhui Province, People's Republic of China; Beijing Institute of Genomics, Chinese Academy of Sciences, China

## Abstract

The metagenomic method directly sequences and analyses genome information from microbial communities. The main computational tasks for metagenomic analyses include taxonomical and functional structure analysis for all genomes in a microbial community (also referred to as a metagenomic sample). With the advancement of Next Generation Sequencing (NGS) techniques, the number of metagenomic samples and the data size for each sample are increasing rapidly. Current metagenomic analysis is both data- and computation- intensive, especially when there are many species in a metagenomic sample, and each has a large number of sequences. As such, metagenomic analyses require extensive computational power. The increasing analytical requirements further augment the challenges for computation analysis. In this work, we have proposed Parallel-META 2.0, a metagenomic analysis software package, to cope with such needs for efficient and fast analyses of taxonomical and functional structures for microbial communities. Parallel-META 2.0 is an extended and improved version of Parallel-META 1.0, which enhances the taxonomical analysis using multiple databases, improves computation efficiency by optimized parallel computing, and supports interactive visualization of results in multiple views. Furthermore, it enables functional analysis for metagenomic samples including short-reads assembly, gene prediction and functional annotation. Therefore, it could provide accurate taxonomical and functional analyses of the metagenomic samples in high-throughput manner and on large scale.

## Background

The total number of microbial cells on earth is huge: approximate estimation of their number is 10^30^ [1], and the genomes of these vastly unknown microbes might contain a large number of novel genes with very important functions. However, more than 99% of microbe species remain unknown, un-isolated or un-culturable [2], making traditional isolation and cultivation process non-applicable. Metagenomics refer to the study of genetic materials recovered directly from environmental samples [3], which has made it possible for better understanding of microbial diversity as well as their functions and interactions. The broad applications of metagenomic research, including environmental sciences, bioenergy research and health care, have made it an increasingly popular research area.

There are two major analysis tasks for metagenomic samples: taxonomical and functional analyses (**Table 1**). For taxonomical analyses, early metagenomic survey of microbial communities focused on 16S ribosomal RNA sequences which are relatively short, often conserved within a species while different between species. The 16S rRNA-based metagenomic survey has already produced data for analysis of microbial communities of Sargasso Sea [4], acid mine drainage biofilm [5], human gut microbiome [6] and so on. Recently, some 16S rRNA amplicon data analysis pipelines were introduced, such as PHYLOSHOP [7], Mothur [8] and QIIME [9]. However, the increasing number of metagenome data analysis projects needs more and more computing power, which becomes an increasingly large huddle for the efficient process of metagenome datasets by current pipelines. The functional analysis of metagenomic data is based on shotgun sequencing data that could elucidate the gene-set, pathway and even regulation network properties and their dynamics for microbial communities. The most frequently used analysis methods for shotgun sequencing data including MEGAN [10], CARMA [11], Sort-ITEM [12], ALLPATHS-LG [13] and IDBA [14] are designed for only part of the functional analysis, such as binning and assembly, cannot complete the whole functional annotation processes. Meanwhile the web-based metagenomic annotation platforms, such as MG-RAST [15] and CAMERA [16], have been designed to analyze metagenomic data for functional annotation. Nevertheless, there are currently few tools that integrate taxonomical and functional analysis of metagenomic samples.

**Table 1:** The comparison of properties of taxonomical and functional analyses for metagenomic samples.

Features	Taxonomical analysis	Functional analysis
**Input data types**	Pyro-sequencing or shotgun sequencing data	shotgun sequencing data
**Basic object sequences**	16S rRNA sequences	Gene sequences
**Reference databases**	GreenGenes, RDP, Silva, etc.	NCBI NR, SEED. etc.
**Basic results**	Taxonomy structure (Qualitative and quantitative)	Functional hierarchy (Qualitative)
**Extended results**	Sample taxonomical structure comparison	Sample functional composition comparison, Functional and pathway enrichment
**Applications**	Novel strain identification	Gene discovery

At present one critical bottleneck in metagenomic analysis is the efficiency of data process because of the slow analysis speed. As metagenomic data analysis task is both data- and computation-intensive, high-performance computing is needed, especially when (1) the dataset size is huge for a sample, (2) a project involves many metagenomic samples and (3) the analyses are complex and time-sensitive. Moreover, the increasing number of metagenomic projects usually requires the comparison of different samples. Yet current methods are limited by their low efficiency [7], [10], [11]. Thus, high-performance computational techniques are needed to speed-up analysis, without compromising the analysis accuracy.

In this work, we have designed Parallel-META 2.0 for taxonomical and functional analysis of metagenomic samples based on High Performance Computing (HPC). Parallel-META 2.0 is the improved version of Parallel-META 1.0 [17] with several significant updates. Firstly, the optimized parallel computing and I/O strategy achieved more than 12 times speed-up compared to PHYLOSHOP [7], 3 times faster than MetaPhlAn [18], and 1.4 times faster than version 1.0. Secondly, in version 2.0, the taxonomical analysis has been enhanced by (a) supporting 18S rRNA extraction & analysis for Eukaryote, (b) extending to multiple reference database annotation and (c) providing taxonomical comparison between multiple samples. Thirdly, Parallel-META 2.0 enables functional analysis based on both GO-term (Gene Ontology term) annotation [19] and SEED [20] annotation methods. Finally, results could be visualized and compared from different angles, rather than the plain text by version 1.0. This visualization of results makes the structures of microbial communities easier for human reading, and thus could simplify in-depth manual analyses.

Parallel-META 2.0 software package is available at http://www.computationalbioenergy.org/parallel-meta.html, which is released under *MIT license*. We have also built a project about this work in GitHub and uploaded the source code, manual, materials and example datasets onto it. The link of Parallel-META 2.0 is at https://github.com/Comp-Bio-Group/Parallel-META. As of now, this Parallel-META software package and the corresponding data analysis platform have supported more than 30 metagenomic data analysis projects around the world.

## Methods

**Figure 1:**
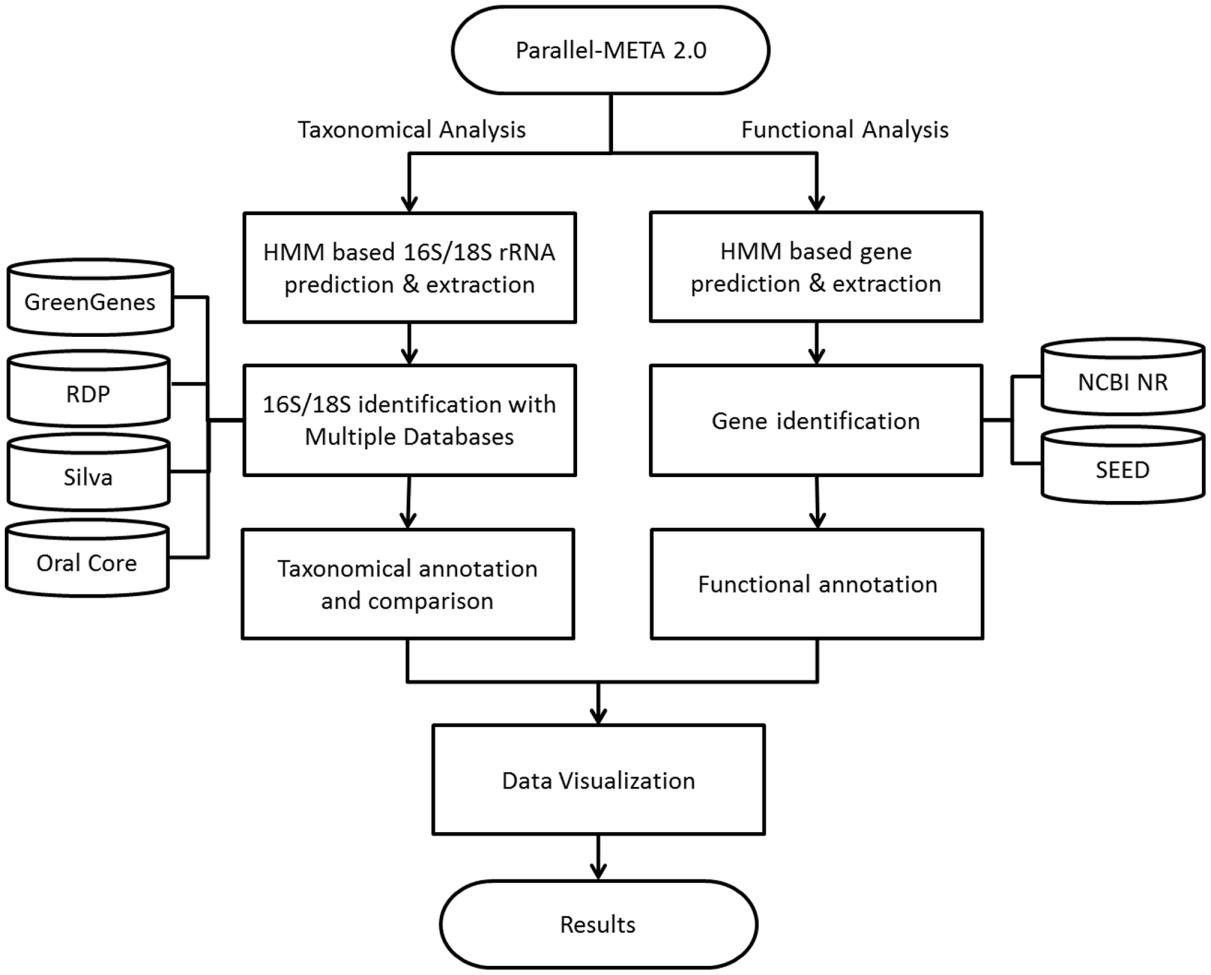
The overall framework of Parallel-META 2.0.

In this work we have developed the Parallel-META 2.0 package to analyze the metagenomic samples by incorporating several novel functions including improved computational engine based on High-Performance Computing (HPC), enhanced methods for taxonomical structure interpretation, functional analysis, as well as the data visualization techniques. The overall framework is illustrated in **Figure 1**.

### High Performance Computing

Metagenomic data analysis is both data- and computation-intensive. To meet the needs for efficient process, we have improved the High-Performance Computing (HPC) of Parallel-META software [14] based on GPU and multi-core CPU which enabled the highly-efficient metagenomic analysis in both taxonomical and functional analyses.

Parallel-META [14] software processes the metagenomic taxonomical analysis with shotgun sequencing data in 3 steps: (1) 16S rRNA extraction: To predict and extract 16S rRNA fragments from the input data by Hidden Markov Model (also referred to as HMM) algorithm [21], [22] with the HMM model built from all 16S rRNA sequences of Silva Database [23]. (2) 16S rRNA mapping: To identify each component of the microbial community by mapping all 16S rRNA fragments to reference database by parallel computing. (3) Taxonomical annotation: To parse out taxonomical and phylogenetic structure of the microbial community.

For highly efficient parallel computing algorithm design and deep optimization, POSIX thread and OpenMP techniques, together with CUDA (Compute Unified Device Architecture) programming techniques based on Linux C++ have been used in the computing engine of Parallel-META 2.0 for 16S/18S rRNA & gene prediction and annotation. In addition, this new version of computing engine also has optimized I/O efficiency, which is one of key features for high performance computation.

#### 1.1 HMM based 16S/18S rRNA and gene prediction

In Parallel-META 2.0, the 16S/18S rRNA fragments and genes are predicted and extracted from the input data based on Hidden Markov Model algorithm [22]. For taxonomical analysis, 16S/18S rRNA fragment extraction is realized by GPU based HMM [21], [24], [25] with parallel computing on GPU, while the gene prediction is realized by FragGeneScan [26].

#### 1.2 Parallel computing for BLAST based sequence mapping

The extracted 16S/18S rRNA fragments and gene sequences from the input data are then mapped to the reference database for identification and annotation, which is a time consuming process. To reduce the time cost of database search, we have implemented the parallel sequence mapping in Parallel-META 2.0 using OpenMP and CUDA. Input reads are divided into smaller sets, and then the mapping task of each set is assigned to independent thread, which could be processed on different processors or cores in parallel.

#### 1.3 Input & Output strategy

16S/18S rRNA sequences exist in both the original reads and the complementary reads of input shotgun sequencing data. After loaded the original reads into hash table of RAM, all computation processes, including the complementary reads transformation, 16S/18S rRNA prediction and extraction, are processed in the RAM rather than extern I/O operation on file system and Hard Disk Drivers (also referred to as HDD).

### Enhanced Taxonomical analysis

Based on computing method of version 1.0 described in section “High Performance Computing” Parallel-META 2.0 improves all 3 steps of taxonomical analyses as below:

#### 2.1 16S/18S rRNA fragment extraction

In addition to that Parallel-META 1.0 can only analyze Bacteria domain by 16S rRNA, In 2.0, Eukaryote domain could also be handled by adding the 18S rRNA extraction by the Hidden Markov Model (HMM) algorithm [22] with HMM model built based on Silva Database [23].

#### 2.2 Multiple databases

Reference databases are used to identify and classify the taxonomical components for the microbial community samples based on mapped 16S/18S rRNA reads. Reference database schemes of Parallel-META 2.0 is an extension from a single database of previous version (1.0 using only GreenGenes [27]) to multi-database including GreenGenes, Silva [23], RDP [28] and Oral Core [29] (**Table 2** shows the sequence number information). Since the accuracy of the metagenomic analysis results heavily depends on the completeness and correctness of reference databases, to this end, multi-database is enabled for 2 reasons: (a) Each database is for specific purpose: the GreenGenes, RDP and Silva databases are considered to be relatively complete yet general, and Oral Core database is specifically designed for species in oral environment with more details; (b) Community structure of metagenomic sample generated based on multiple databases can also be integrated to mitigate the bias and incompleteness caused by using single reference database.

**Table 2:** Information about multiple 16S rRNA reference databases.

Reference Database	# of Sequence	Utilization in Parallel-META
**GreenGenes**	4939	1.0 and 2.0
**RDP**	8423	2.0 only
**Silva**	14975	2.0 only
**Oral Core**	1046	2.0 only

#### 2.3 Multi-sample comparison

Parallel-META 2.0 contains new module to qualitatively and quantitatively compare the taxonomical structures among metagenomic samples on different biology levels (from domain level to species level). The comparison results could also be elucidated in the “global view” and “sub-sample view” described in section “Interactive visualization”.

### Functional analysis

**Figure 2:**
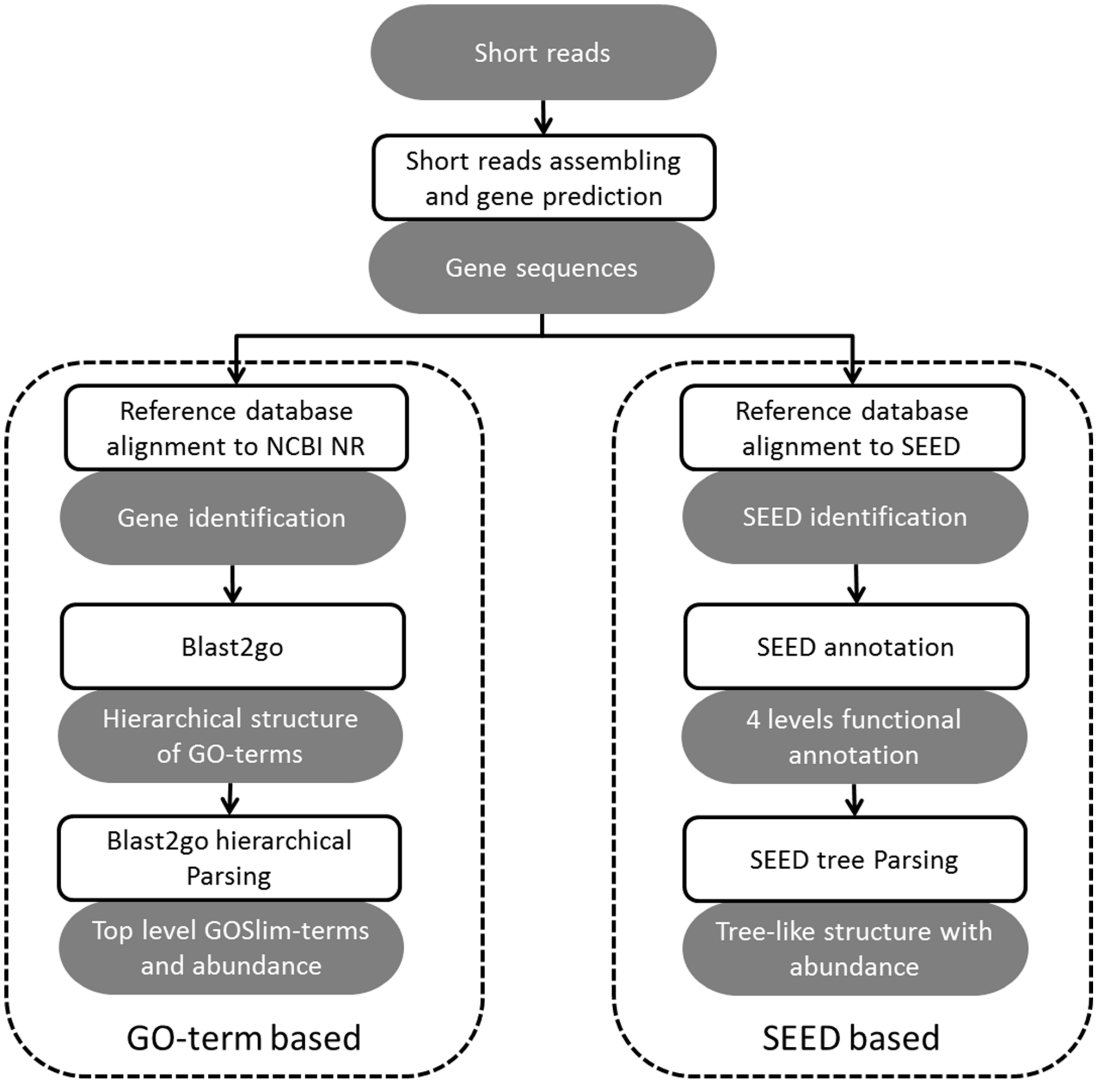
The dual computational engines for functional analysis in Parallel-META 2.0.

In Parallel-META 2.0, we enable functional analysis of metagenomic samples based on two computational engines: one based on GO-term annotation, another based on SEED annotation, both of which can be divided into 3 steps (**Figure 2**): (1) short-reads assembly and gene prediction, (2) gene identification and (3) functional annotation followed by functional structures interpretation of microbial communities.

#### 3.1 Short-reads assembly and gene prediction

Short reads in shotgun sequencing data from input metagenomic samples need to be assembled into longer contigs for gene sequence prediction. Here Velvet [30] is chosen as the short reads assembler which plays the role as a module in Parallel-META 2.0, and then gene sequences are predicted based on Hidden Markov Model (HMM) algorithm [22], [26]. Note that although there are some other assembly tools such as ALLPATH-LG [13] that could outperform Velvet, we have still used Velvet assembler in this study as it is widely used in the area.

#### 3.2 Gene identification

All predicted gene sequences from the assembled contigs are then mapped to the reference database for their identification. In Parallel-META 2.0, the gene identification is implemented by two computational engines with different reference database: GO-term based that maps gene sequences to NCBI NR database (http://www.ncbi.nlm.nih.gov/), and SEED based that maps gene sequences to SEED [16] database. As the database alignment is time consuming, this process is parallelized to achieve high efficiency (refer to section 1.2 for details).

#### 3.3 Functional annotation

Two kinds of methods are used in functional annotation as gene identification, GO-term based and SEED based. For GO-term based analysis, we use blast2go [15] to get all the GO-terms in the sample. GOSlim-terms (manually curated by experts, OBO version 1.2, http://www.geneontology.org/GO_slims/goslim_generic.obo) with important biological meanings would be our focus. All GO-terms are tracked to higher levels to parse out the GOSlim-terms, and most abundant GOSlim-terms are then extracted with abundance values. For SEED based analysis, each gene sequence can be assigned to exactly 4 levels of SEED [20] annotations. This tree-like functional structure with abundance values can be visualized in the same way as the taxonomical structure (refer to section 4.2 for details).

### Interactive visualization

**Figure 3:**
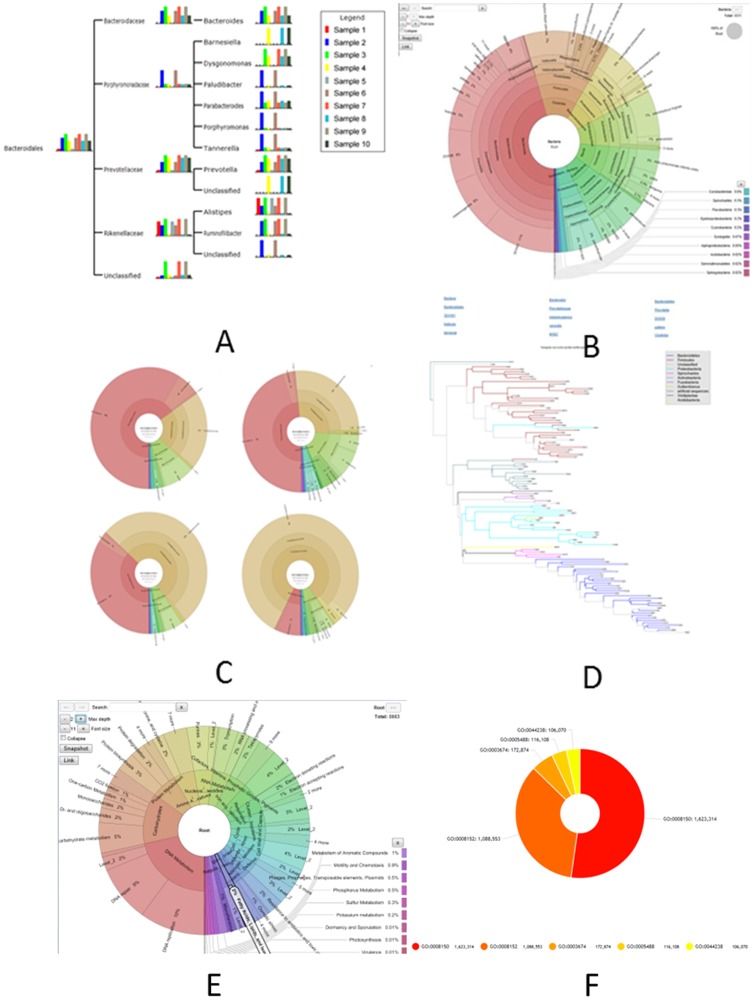
The visualization effects for different kinds of results. (A) Global view, (B) Sample view, (C) Sub-sample view and (D) Phylogenetic view, (E) Sample view for SEED based functional analysis, and (F) GO-term based analysis.

We have used several visualization techniques, such as HTML5, SVG and JavaScript, in Parallel-META 2.0 for analytical result visualization (**Figure 3**), which could either be browsed by online visualization tool MetaSee [31], or by standalone web browsers. Based on these techniques, Parallel-META 2.0 supports (a) interactive visualization of the taxonomical & functional community structure (b) smooth change among different angles for the same sample and (c) comparison of different samples in a single interface. These visualization functionalities are described as below:

#### 4.1 Taxonomical structure visualization

Taxonomical visualization shows the taxonomical structure of a microbial community in the following angles:

(1) Global view is a taxonomical hierarchical tree that contains all taxa and their proportions in single or multiple samples. Thus it shows the whole profile with all samples been compared (**Figure 3 (A)**). (2) Sample view represents the community taxonomical structure in a dynamic multi-layer pie-chart, which also supports smooth shift among multi-sample comparisons of their taxonomical community structures (**Figure 3 (B)**). The sample view is implemented partly by the Krona software [32]. (3) Sub-sample view is a detailed “sample view” that focuses on one node (a specific taxa) in the “global view” taxonomical hierarchical tree structure, which is useful for comparing different samples for a specific taxa (**Figure 3 (C)**). (4) Phylogenetic view is a phylogenetic tree based on 16S rRNA reads from the metagenomic sample data, which elucidates the evolutionary relationship of all microbes in a microbial community sample (**Figure 3 (D)**).

#### 4.2 Functional structure visualization

Functional visualization reflects the functional structure of a microbial community. For SEED based functional analysis, as the microbial community structure can be parsed into 4-level-annotations, this hierarchal annotation structure could be visualized in the same way as the taxonomical visualization in “Sample view” (**Figure 3 (E)**). For GO-term based analysis, the GO-terms are illustrated into a pie-chart containing each function's proportional information using SVG (**Figure 3 (F)**)..

## Results

### Datasets and Experiment design

In this work we have evaluated the performance of Parallel-META 2.0 in taxonomical and functional analysis based on both simulated datasets and real datasets. All experiments were completed on a desktop sever with dual Intel Xeon X5650 (12 cores, supporting 24 threads in total), 72 GB RAM and NVIDIA Tesla C2075 GPU (448 processors, 6 GB on board memory).

For the real metagenomic data, we have collected 3 human saliva metagenomic samples [17] and 3 human gut samples [33] from different people (**Table 3**). Saliva samples were sequenced by Illumina Solexa GA-IIx into pair-end 100 bp reads, and sequences of gut samples were produced by 454 FLX.

**Table 3:** Information about real metagenomic datasets.

Dataset	Biological background	Source	Sequencer	# of Reads	Size (MB)
**Real Sample 1**	Saliva sample, person 1, healthy	MG-RAST, 4454806.3	Illunima Solexa	17,591,235	1576.96
**Real Sample 2**	Saliva sample, person 2, decayed tooth	MG-RAST, 4454817.3	Illunima Solexa	34,405,667	2775.04
**Real Sample 3**	Saliva sample, person 3, decayed tooth	MG-RAST, 4454816.3	Illunima Solexa	28,854,628	2928.64
**Real Sample 4**	Gut sample, person 4	CAMERA2, TS3_SRX001344	454 FLX	510,972	198.70
**Real Sample 5**	Gut sample, person 5	CAMERA2, TS50_SRX001358	454 FLX	549,700	216.76
**Real Sample 6**	Gut sample, person 6	CAMERA2, TS7_SRX001348	454 FLX	555,853	238.35

The simulated datasets contained 10 samples, which were constructed based on human oral related microbial genomes from HOMD database [34], with *Fusobacterium periodonticum, Veillonella dispar, Porphyromonas gingivalis, Prevotella tannerae, Veillonella sp., Rothia dentocariosa, Actinomyces odontolyticus, Megasphaera micronuciformis* etc. as most abundant species. All simulated datasets were created by metagenomic data simulator Dwgsim (Whole Genome Simulation. http://sourceforge.net/apps/mediawiki/dnaa/index.php?title=Whole_Genome_Simulation) based on randomly selected genomes above with suitable abundances (most abundant 2∼3 species are manually defined, while others are defined randomly). And these genome sequences were translated into short reads with 100 bp length and 1% sequencing errors. **Table 4** illustrated the basic information of simulated datasets, and the detail information including genome names and abundance value are available in supplementary materials (**Table S1 (A)–(J)** in **File S1**).

**Table 4:** Information about simulated metagenomic datasets.

Dataset	# of Reads	Size (MB)	# of Strains
**Simulated Sample 1**	18,936,022	2,180.02	10
**Simulated Sample 2**	21,860,962	2,517.56	14
**Simulated Sample 3**	17,702,336	2,042.55	11
**Simulated Sample 4**	21,850,592	2,518.66	13
**Simulated Sample 5**	17,003,492	1,955.71	10
**Simulated Sample 6**	20,156,006	2,328.32	12
**Simulated Sample 7**	19,700,952	2,270.77	11
**Simulated Sample 8**	23,718,286	2,732.70	13
**Simulated Sample 9**	21,6,68,126	2,498.10	13
**Simulated Sample 10**	20,345,442	2,337.97	11

Refer to **Table S1** (**A**)–(**J**) in **File S1** for detailed strains and their relative abundances for these simulated samples.

### Efficiency evaluation

**Figure 4:**
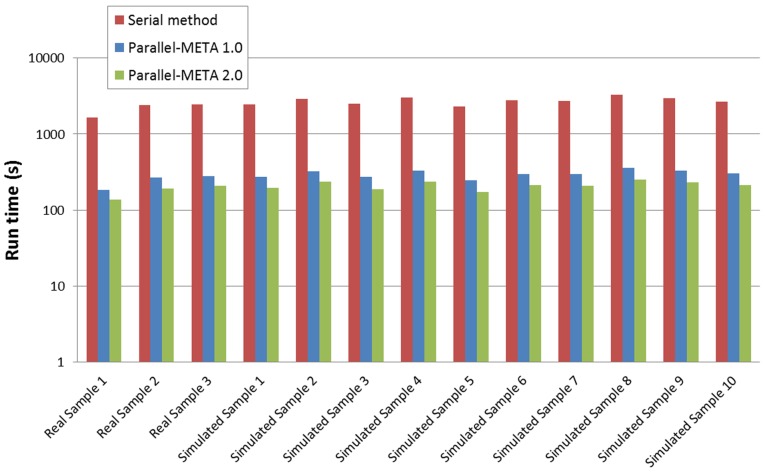
Running time of Parallel-META 1.0 and 2.0 with the same datasets and reference database (Greengenes). The Y-axis is in 10-based log scale.

With the improved High Performance Computing strategy, the efficiency of Parallel-META 2.0 could be significantly increased. In this part we compared the two version of Parallel-META, as well as two other methods (PHYLOSHOP [7] and MetaPhlAn [18]) based on 3 real saliva samples in **Table 3** (real sample 1, 2 and 3) and all 10 simulated samples in **Table 4**. All tests were performed on the same hardware platform to evaluate the acceleration in running time (refer to **Table S2** in **File S1** for detailed parameter configuration). From results in **Figure 4** we could see that in the same condition, version 2.0 could achieve an average speed up of 12.76 to PHYLOSHOP, 3.01 to MetaPhlAn, and 1.41 to Parallel-META version 1.0. This advantage in processing efficiency would enable the in-depth analysis among massive metagenomic data. In addition, there was no significant difference between real samples and simulated samples on process efficiency (**Figure 4**).

### Taxonomical Analysis and Visualization

#### Error rate test with simulated data

In this experiment, we tested the error rate of taxonomical analysis on both phylum and genus level by using 10 simulated datasets (**Table 4**). We defined the error rate for taxonomical analysis as below:

Assume that there were *N* taxa (*T_i_*, i = 0 to N-1) at a specified taxonomical level (phylum or genus) in a simulated sample. *V_i_* was the abundance value of taxa *T_i_* in the simulated data, while *V′_i_* was the abundance value in the analysis result for taxa *T_i_*.

Then for this simulated data, the error rate E could be calculated as
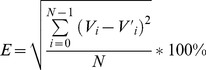
(1)which essentially calculated the Euclidean distance between predicted and actual taxonomical structures of the microbial community. Therefore, larger *E* value indicates higher error rate.

Currently, taxonomical analysis of Parallel-META 2.0 has been enhanced by using multiple reference databases including GeneGenes, RDP, Silva and Oral Core, rather than the single reference database of GreenGenes in version 1.0. In this work results by Parallel-META 1.0 with database GreenGenes were considered as “control results”, and the comparison among results based on different databases was conducted to show the improved accuracy of Parallel-META 2.0 at both phylum level genus level using **Formula (1)**.

**Figure 5:**
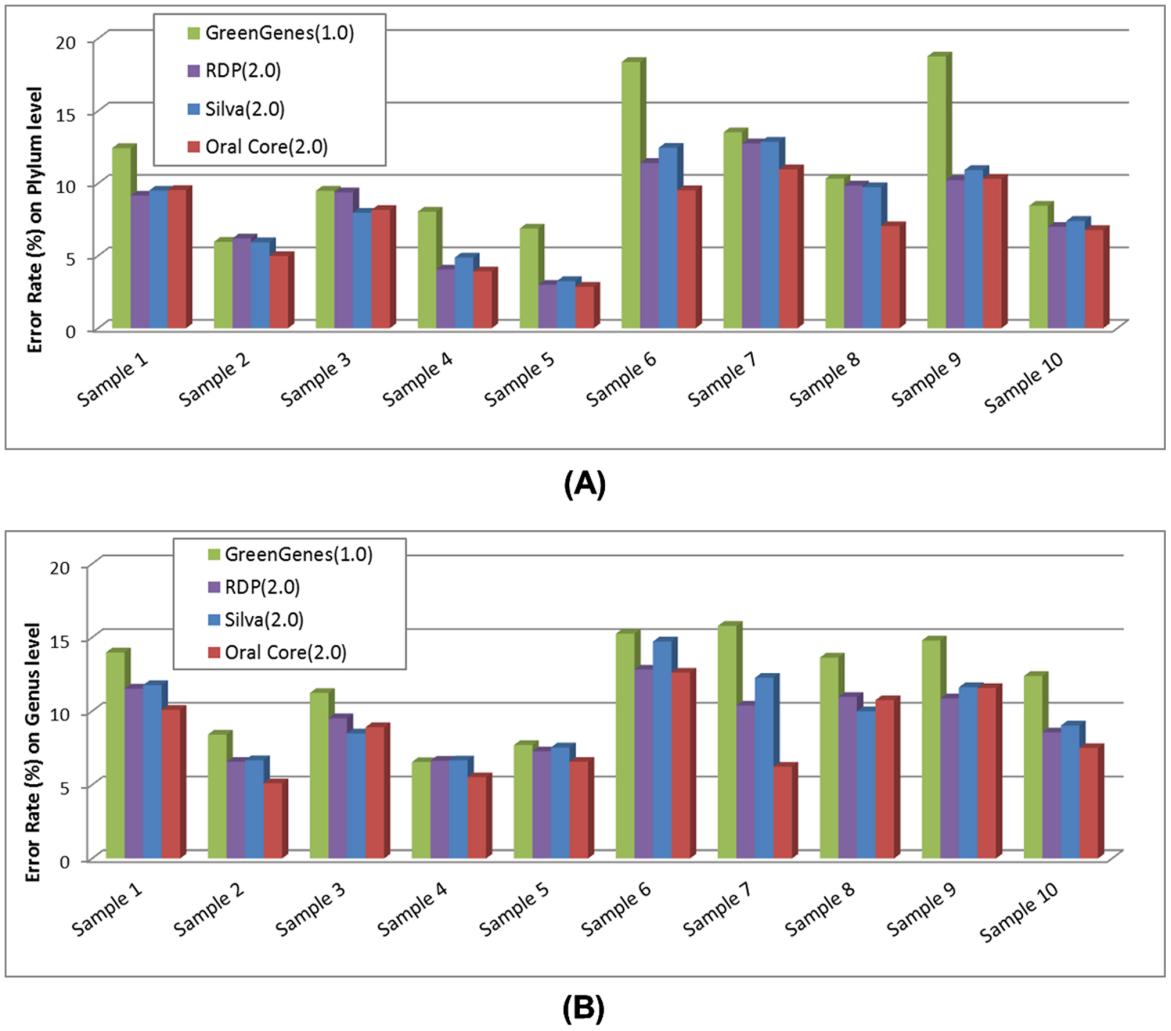
Error rate of taxonomical analysis of Parallel-META 1.0 and 2.0 with simulated data based on different databases at (A) phylum level and (B) genus level.

The error rates were illustrated in **Figure 5**. From the results we could observe that the error rates of taxonomical analysis at phylum and genus levels could be reduced with the extended sets of reference databases. In **Table 5** the average error rates of 7.40% at phylum level and 8.48% at genus level also indicated the reliability of the improved taxonomical analysis of Parallel-META 2.0. In addition, as all species of the simulated samples were from human oral microbial genomes (**Table S1** in **File S1**), results based on the reference database Oral Core could achieve the lowest error rate benefited from the optimized annotations of oral environment microbes.

**Table 5:** Average error rate of taxonomical analysis of Parallel-META 2.0.

Reference Database	Average error rate (%) at Phylum level	Average error rate (%) at Genus level
**GreenGenes**	11.22	11.97
**RDP**	8.30	9.51
**Sliva**	8.49	9.87
**Oral Core**	7.40	8.48

#### Multiple Sample Comparison and Visualization

**Figure 6:**
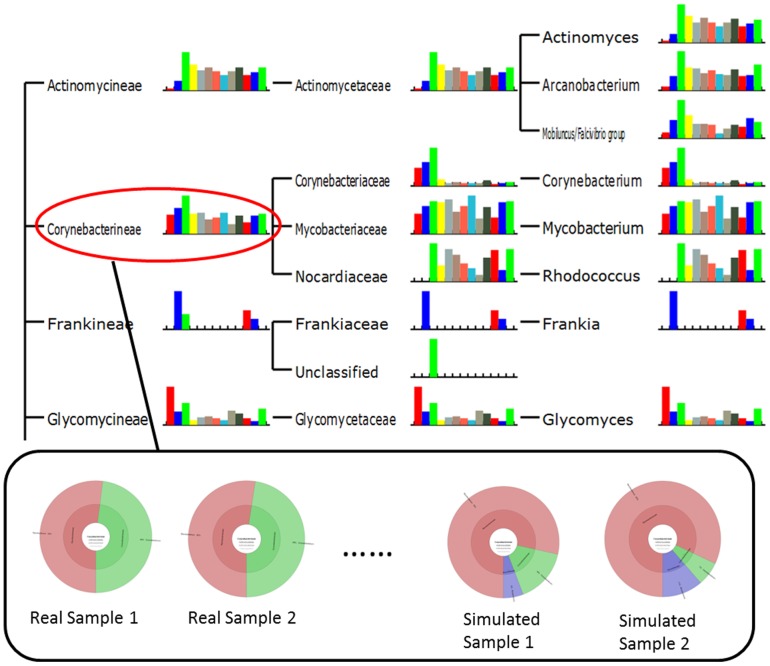
Multiple sample comparison by Parallel-META 2.0.

We also compared the taxonomical structure of 3 real saliva datasets (**Table 3**) and 10 simulated datasets (**Table 4**) by Parallel-META 2.0. All datasets' community structures were drawn into one tree-like global view (refer to section 4.1 in “Methods” for details) for comparison. Abundance values were illustrated in bar-chart of the global view (**Figure 6**). Besides, sub-sample view of all samples could also be linked by the corresponding taxa names in the tree.

Among the 3 real saliva samples, we have found that the most abundant taxa in sample 1 (healthy saliva sample) included *Flavobacteria*, while the most abundant taxa in sample 2 and 3 (decayed saliva samples) included *Clostridia* and *Bacillales*. These results showed that there was a significant difference in most abundant taxa when comparing healthy and decayed saliva metagenomic samples, indicating that the microbial community structures could represent oral health status of the hosts [35].

### Functional Analysis

**Figure 7:**
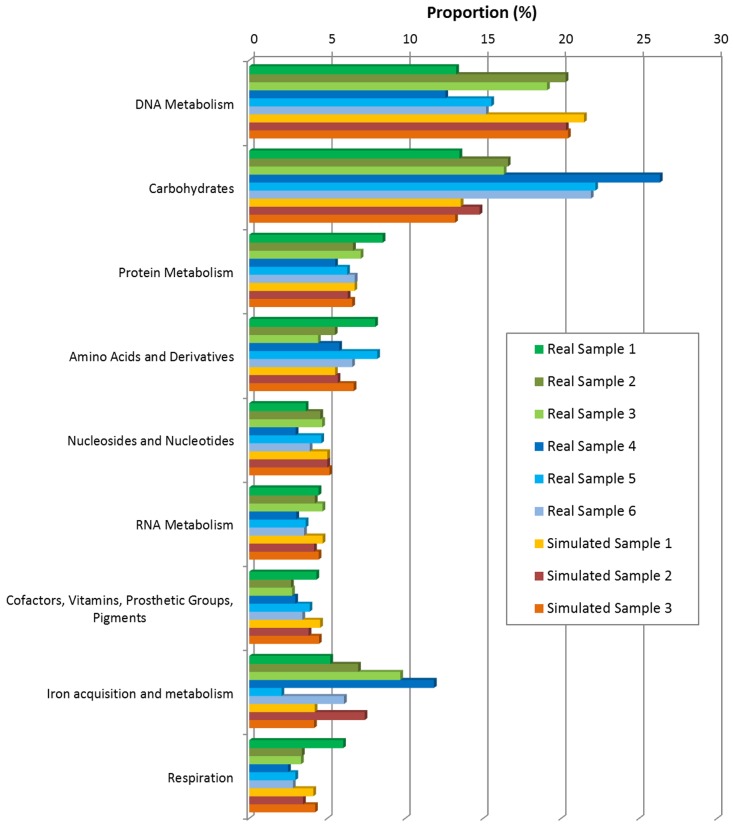
Functional annotation results by GO-term based method.

**Figure 8:**
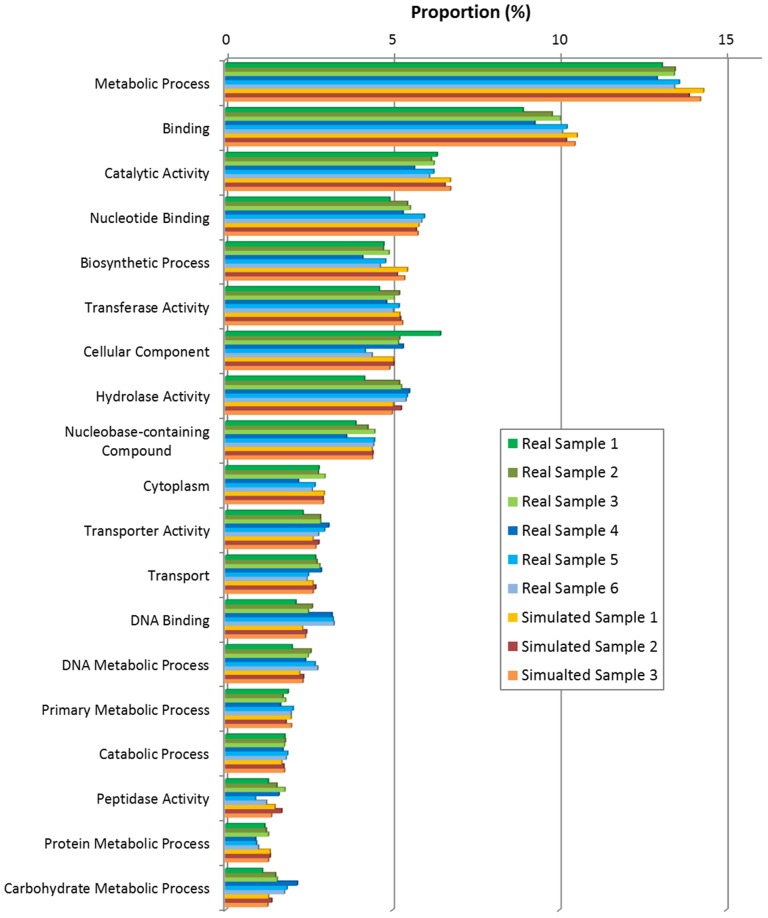
Functional annotation results by SEED based method.

In this part, all 6 real samples (saliva and gut samples in **Table 3**) and 3 simulated samples (simulated sample 1, 2 and 3 of **Table 4**) were used to test both GO-term based and SEED based functional analysis. Results in **Table 6**, **Figure 7** and **Figure 8** (also refer to supplementary materials **Table S3** and **Table S4** in **File S1** for detailed statistical information) have shown the functional structure patterns of these samples as below:

**Table 6:** Statistical results of Parallel-META 2.0 functional analysis.

Dataset	# of Read contributing to Predicted Gene	# of GOSlim terms	# of Predicted Genes (from SEED)
**Real Sample 1**	17,591,235	21,975	1,462
**Real Sample 2**	28,854,628	933,398	27,927
**Real Sample 3**	34,405,667	467,332	22,833
**Real Sample 4**	510,972	318,408	19,640
**Real Sample 5**	549,700	262,887	15,099
**Real Sample 6**	555,853	350,125	21,488
**Simulated Sample 1**	18,936,022	182,120	8,664
**Simulated Sample 2**	21,860,962	277,897	14,046
**Simulated Sample 3**	17,702,336	199,097	10,126

Refer to **Table S3** and **Table S4** in **File S1** for details of results on these samples. Notice that as many of the speciesd in real samples do not have complete genome sequences, the expected number of genes that could be predicted is hard to estimate for these samples.

1. Firstly, results showed that in GO-term based analysis (**Figure 7** **, Table S3** in **File S1**), the most abundant terms of both real and simulated data from oral environment were consistently *Metabolic Process, Biosynthetic Process, DNA metabolic process, etc.*, for Biological Process, *DNA binding, Catalytic Activity, Nucleotide Binding, etc.*, for Molecular Function, and *Cytoplasm* for Cellular Component. This indicated that the simulated data was similar to real data as regard to functional profile. However, the fact that there exist larger variations among real samples than those among simulated samples indirectly proved the needs for real data: the variation in functional profiles could not yet be fully characterized by simulated data only. In SEED based analysis results (**Figure 8** **, Table S4** in **File S1**), the most abundant functions of both real and simulated data from oral environment included *DNA metabolism*, *Carbohydrates* and *Protein Metabolism*, showing high consistency between real samples and simulated samples.
2. Secondly, real samples from gut environment (Real sample 4, 5, 6) had *Carbohydrates, DNA metabolism, Iron acquisition and metabolism and Amino Acids and Derivatives* as most abundant functions, which had also been observed in *Peter J. Turnbaugh et al., 2009* [33].
3. Thirdly, functional profiles of simulated samples (Simulated sample 1, 2, 3) were more similar to real samples (Real sample 1, 2, 3) from oral environment than gut environment samples (Real sample 4, 5, 6), considering both GO-termed based and SEED based annotations, especially in *DNA Metabolic process, Catalytic Activity, Biosynthetic Process* for GO-term based annotation, and *DNA Metabolism, Carbohydrates, Nucleosides and Nucleotides* for SEED based annotation. These results also elucidated the consistency between real and simulated datasets, as well as that between different annotation methods.

## Conclusion and Discussions

In this work, we have proposed Parallel-META 2.0, a powerful metagenomic analysis package based on the joint-force of (a) advanced and comprehensive methods/databases for taxonomical and functional analysis of metagenomic samples, (b) High Performance Computing and (c) cutting-edge interactive visualization technique.

Our preliminary tests on both simulated and real datasets have shown that this software is not only accurate and fast, but also with significantly improved user experience to facilitate in-depth analysis and comparison of microbial community samples.

1. Overall computing performance. Compared to version 1.0, Parallel-META 2.0 has enhanced computing engine in 16S/18S rRNA and gene prediction and sequence alignment module by in-depth optimization in programming and algorithm implementation. Moreover, combined with the improved I/O strategy, Parallel-META 2.0 already has obvious progress in computing speed compared to the previous version. As currently the HDD I/O bandwidth via SATA bus is a bottleneck in the system, I/O throughput could be enhanced significantly by Solid State Disk.
2. Improved taxonomical annotation based on multiple databases. New version of Parallel-META provides a wider range with 16S and 18S rRNA extraction and analysis for Bacteria and Eukaryote domains, and multiple reference databases for better analysis precision, as well as suitable for specialized users. In addition, multiple-sample comparison enables the identification of common structure among microbial communities.
3. Functional annotation of metagenomic samples. Parallel-META 2.0 has included functional annotation modules, so that it could cover a more complete area of metagenomic data analysis. With the importance of functional interpretation of metagenome been understood, as well as more and more WGS (Whole Genome Sequencing) metagenomic sample datasets been generated, these analyses would become increasingly important.
4. Visualization of analysis results. Users experience could be largely improved by the friendly displayed interactive graphs from various angles of the microbial communities, including the global taxonomical structure, comparison on specified taxonomical levels, phylogenetic relationships, GO-term based and SEED based annotations.

As such, Parallel-META 2.0 has already served for more than 30 research groups worldwide. And it can be foreseen that this software could benefit an even broader area of metagenomic research.

Current Parallel-META is a stand-alone method for analysis of taxonomical and functional structure of the metagenomic samples. The efficiency and throughput can be reinforced by clouding computing in further works. On a broader scale, Parallel-META 2.0 could be combined with metagenomic database for more in-depth and large-scale data-mining for analysis of metagenomic samples, by providing efficient method for extracting and comparing taxonomical or functional features of microbial communities.

## Supporting Information

File S1 **Supplementary files Tables S1–S4 for support information.**
(DOCX)
