# Supplementary material for: Parallel-META 2.0: Enhanced Metagenomic Data Analysis with Functional Annotation, High Performance Computing and Advanced Visualization
Source: PLoS One. 2014 Mar 3;9(3):e89323. doi: 10.1371/journal.pone.0089323 (PMC3940597; doi:10.1371/journal.pone.0089323)
Supplement: File S1 — Supplementary files Tables S1–S4 for support information. (DOCX) [file pone.0089323.s001.docx]

Supplementary of “Parallel-META 2.0: Enhanced Metagenomic Data Analysis with Functional Annotation, High Performance Computing and Advanced Visualization”

# Tables

**Table S1. Structure of Simulated Samples**

**(A) Simulated Sample 1**

| **Genome Name** | **Relative Abundance(%)** |
| --- | --- |
| Fusobacterium periodonticum ATCC 33693 | 0.241413 |
| Veillonella dispar ATCC 17748 | 0.199884 |
| Porphyromonas gingivalis ATCC 33277 | 0.111474 |
| Prevotella tannerae ATCC 51259 | 0.081416 |
| Veillonella sp. oral taxon 158 str. F0412 | 0.034348 |
| Prevotella denticola F0289 | 0.092705 |
| Rothia dentocariosa ATCC 17931 | 0.039543 |
| Actinomyces odontolyticus ATCC 17982 | 0.075549 |
| Megasphaera micronuciformis F0359 | 0.055475 |
| Veillonella parvula ATCC 17745 | 0.068193 |

**(B) Simulated Sample 2**

| **Genome Name** | **Relative Abundance (%)** |
| --- | --- |
| Prevotella denticola F0289 | 0.233064041 |
| Fusobacterium periodonticum ATCC 33693 | 0.168590454 |
| Porphyromonas endodontalis ATCC 35406 | 0.109070509 |
| Rothia dentocariosa ATCC 17931 | 0.066275002 |
| Megasphaera micronuciformis F0359 | 0.023244555 |
| Veillonella dispar ATCC 17748 | 0.055835508 |
| Prevotella veroralis F0319 | 0.039144997 |
| Veillonella parvula ATCC 17745 | 0.057147066 |
| Campylobacter rectus RM3267 | 0.066223822 |
| Porphyromonas gingivalis ATCC 33277 | 0.031139129 |
| Actinomyces odontolyticus ATCC 17982 | 0.031655705 |
| Streptococcus peroris ATCC 700780 genomic | 0.021597083 |
| Veillonella sp. oral taxon 158 str. F0412 | 0.028784283 |
| Prevotella tannerae ATCC 51259 | 0.068227846 |

**(C) Simulated Sample 3**

| **Genome Name** | **Relative Abundance (%)** |
| --- | --- |
| Porphyromonas gingivalis ATCC 33277 | 0.193516 |
| Prevotella tannerae ATCC 51259 | 0.212003 |
| Campylobacter rectus RM3267 | 0.164621 |
| Streptococcus peroris ATCC 700780 genomic | 0.026843 |
| Prevotella denticola F0289 | 0.096559 |
| Veillonella parvula ATCC 17745 | 0.035514 |
| Megasphaera micronuciformis F0359 | 0.028891 |
| Prevotella veroralis F0319 | 0.097307 |
| Porphyromonas endodontalis ATCC 35406 | 0.067782 |
| Rothia dentocariosa ATCC 17931 | 0.041187 |
| Veillonella sp. oral taxon 158 str. F0412 | 0.035776 |

**(D) Simulated Sample 4**

| **Genome Name** | **Relative Abundance (%)** |
| --- | --- |
| Porphyromonas endodontalis ATCC 35406 | 0.155467 |
| Rothia dentocariosa ATCC 17931 | 0.226721 |
| Streptococcus peroris ATCC 700780 genomic | 0.123136 |
| Veillonella dispar ATCC 17748 | 0.031835 |
| Veillonella sp. oral taxon 158 str. F0412 | 0.065646 |
| Prevotella tannerae ATCC 51259 | 0.0778 |
| Prevotella veroralis F0319 | 0.044637 |
| Porphyromonas gingivalis ATCC 33277 | 0.071016 |
| Veillonella parvula ATCC 17745 | 0.032582 |
| Actinomyces odontolyticus ATCC 17982 | 0.036097 |
| Campylobacter rectus RM3267 | 0.037758 |
| Prevotella denticola F0289 | 0.044294 |
| Megasphaera micronuciformis F0359 | 0.053012 |

**(E) Simulated Sample 5**

| **Genome Name** | **Relative Abundance (%)** |
| --- | --- |
| Prevotella veroralis F0319 | 0.252652 |
| Porphyromonas gingivalis ATCC 33277 | 0.241176 |
| Campylobacter rectus RM3267 | 0.128228 |
| Actinomyces odontolyticus ATCC 17982 | 0.081726 |
| Porphyromonas endodontalis ATCC 35406 | 0.035198 |
| Veillonella dispar ATCC 17748 | 0.072075 |
| Streptococcus peroris ATCC 700780 genomic | 0.027879 |
| Prevotella denticola F0289 | 0.050142 |
| Veillonella parvula ATCC 17745 | 0.073768 |
| Veillonella sp. oral taxon 158 str. F0412 | 0.037156 |

**(F) Simulated Sample 6**

| **Genome Name** | **Relative Abundance (%)** |
| --- | --- |
| Prevotella denticola F0289 | 0.218899 |
| Actinomyces odontolyticus ATCC 17982 | 0.17839 |
| Rothia dentocariosa ATCC 17931 | 0.186741 |
| Veillonella dispar ATCC 17748 | 0.06293 |
| Prevotella tannerae ATCC 51259 | 0.038449 |
| Campylobacter rectus RM3267 | 0.037319 |
| Fusobacterium periodonticum ATCC 33693 | 0.038003 |
| Veillonella sp. oral taxon 158 str. F0412 | 0.064884 |
| Porphyromonas gingivalis ATCC 33277 | 0.035096 |
| Megasphaera micronuciformis F0359 | 0.026198 |
| Streptococcus peroris ATCC 700780 genomic | 0.048683 |
| Veillonella parvula ATCC 17745 | 0.064409 |

**(G) Simulated Sample 7**

| **Genome Name** | **Relative Abundance (%)** |
| --- | --- |
| Megasphaera micronuciformis F0359 | 0.136909 |
| Fusobacterium periodonticum ATCC 33693 | 0.198597 |
| Actinomyces odontolyticus ATCC 17982 | 0.18645 |
| Rothia dentocariosa ATCC 17931 | 0.039035 |
| Porphyromonas gingivalis ATCC 33277 | 0.036681 |
| Prevotella tannerae ATCC 51259 | 0.080371 |
| Prevotella veroralis F0319 | 0.092224 |
| Veillonella dispar ATCC 17748 | 0.065773 |
| Prevotella denticola F0289 | 0.045758 |
| Veillonella parvula ATCC 17745 | 0.067318 |
| Streptococcus peroris ATCC 700780 genomic | 0.050882 |

**(H) Simulated Sample 8**

| **Genome Name** | **Relative Abundance (%)** | |
| --- | --- | --- |
| Prevotella denticola F0289 | 0.213653 |  |
| Rothia dentocariosa ATCC 17931 | 0.182265 |  |
| Prevotella veroralis F0319 | 0.179423 |  |
| Porphyromonas endodontalis ATCC 35406 | 0.024997 |  |
| Porphyromonas gingivalis ATCC 33277 | 0.057091 |  |
| Streptococcus peroris ATCC 700780 genomic | 0.019798 |  |
| Megasphaera micronuciformis F0359 | 0.042617 |  |
| Veillonella parvula ATCC 17745 | 0.052387 |  |
| Fusobacterium periodonticum ATCC 33693 | 0.03091 |  |
| Prevotella tannerae ATCC 51259 | 0.062545 |  |
| Veillonella sp. oral taxon 158 str. F0412 | 0.052774 |  |
| Veillonella dispar ATCC 17748 | 0.051185 |  |
| Campylobacter rectus RM3267 | 0.030354 |  |

**(I) Simulated Sample 9**

| **Genome Name** | **Relative Abundance (%)** |
| --- | --- |
| Veillonella sp. oral taxon 158 str. F0412 | 0.160771 |
| Veillonella parvula ATCC 17745 | 0.159593 |
| Porphyromonas endodontalis ATCC 35406 | 0.1523 |
| Veillonella dispar ATCC 17748 | 0.062372 |
| Prevotella veroralis F0319 | 0.043728 |
| Streptococcus peroris ATCC 700780 genomic | 0.048251 |
| Fusobacterium periodonticum ATCC 33693 | 0.037666 |
| Prevotella tannerae ATCC 51259 | 0.076215 |
| Campylobacter rectus RM3267 | 0.073977 |
| Actinomyces odontolyticus ATCC 17982 | 0.035362 |
| Prevotella denticola F0289 | 0.086783 |
| Rothia dentocariosa ATCC 17931 | 0.037017 |
| Megasphaera micronuciformis F0359 | 0.025966 |

**(J) Simulated Sample 10**

| **Genome Name** | **Relative Abundance (%)** |
| --- | --- |
| Veillonella dispar ATCC 17748 | 0.191324 |
| Prevotella tannerae ATCC 51259 | 0.194822 |
| Veillonella sp. oral taxon 158 str. F0412 | 0.098631 |
| Prevotella veroralis F0319 | 0.089422 |
| Porphyromonas gingivalis ATCC 33277 | 0.071133 |
| Actinomyces odontolyticus ATCC 17982 | 0.072313 |
| Streptococcus peroris ATCC 700780 genomic | 0.024668 |
| Fusobacterium periodonticum ATCC 33693 | 0.077025 |
| Veillonella parvula ATCC 17745 | 0.065273 |
| Porphyromonas endodontalis ATCC 35406 | 0.062289 |
| Megasphaera micronuciformis F0359 | 0.053099 |

**Table S2. Parameters configuration for the efficient test**

| **Parameter** | **Parallel-META 1.0 & 2.0** | **PHYLOSHOP** | **MetaPhlAn** |
| --- | --- | --- | --- |
| **Reference Database** | GreenGenes | GreenGenes | IMG |
| **Mapping Method** | megablast | megablast | blastn |
| **Mapping E-value** | 1E-10 | 1E-10 | 1E-10 |
| **Core Number** | 12 | 1* | 12 |

*Multi-core is not supported

**Table S3. The most abundant GO-term based functional annotations of (A) real samples from human saliva environment, (B) real samples from human gut environment and (C) simulated samples**

**(A)**

| **Ontology** | **GO-term ID** | **GO-term based Functioinal Annotation** | **Real Sample 1** | | **Real Sample 2** | | **Real Sample 3** | |
| --- | --- | --- | --- | --- | --- | --- | --- | --- |
|  |  |  | Proportion (%) | Count | Proportion (%) | Count | Proportion (%) | Count |
| Biological Process | GO:0008152 | Metabolic Process | 13.08 | 3,308 | 13.47 | 12,5731 | 13.45 | 62,849 |
|  | GO:0009058 | Biosynthetic Process | 4.74 | 1,199 | 4.73 | 44,120 | 4.90 | 22,905 |
|  | GO:0006139 | Nucleobase-containing Compound Metabolic Process | 3.90 | 987 | 4.27 | 39,820 | 4.47 | 20,893 |
|  | GO:0006810 | Transport | 2.70 | 682 | 2.74 | 25,565 | 2.83 | 13,211 |
|  | GO:0050789 | Regulation of Biological Process | 2.57 | 650 | 1.64 | 15,301 | 1.33 | 6,210 |
|  | GO:0006259 | DNA Metabolic Process | 2.00 | 505 | 2.56 | 23,913 | 2.48 | 11,594 |
|  | GO:0044238 | Primary Metabolic Process | 1.88 | 475 | 1.72 | 16,068 | 1.80 | 8,411 |
|  | GO:0009056 | Catabolic Process | 1.78 | 449 | 1.79 | 16,731 | 1.77 | 8,268 |
|  | GO:0019538 | Protein Metabolic Process | 1.17 | 297 | 1.22 | 11,398 | 1.29 | 6,013 |
| Cellular Component | GO:0005575 | Cellular Component | 6.44 | 1,629 | 5.22 | 48,710 | 5.18 | 24,210 |
|  | GO:0005737 | Cytoplasm | 2.80 | 708 | 2.78 | 25,951 | 2.98 | 13,928 |
|  | GO:0005886 | Plasma Membrane | 1.56 | 394 | 1.36 | 12,690 | 1.46 | 6,836 |
| Molecular Function | GO:0005488 | Binding | 8.92 | 2,254 | 9.79 | 91,376 | 10.04 | 46,899 |
|  | GO:0003824 | Catalytic Activity | 6.34 | 1,603 | 6.17 | 57,585 | 6.25 | 29,209 |
|  | GO:0000166 | Nucleotide Binding | 4.92 | 1,245 | 5.45 | 50,866 | 5.54 | 25,883 |
|  | GO:0016740 | Transferase Activity | 4.61 | 1,165 | 5.21 | 48,633 | 5.06 | 23,625 |
|  | GO:0016787 | Hydrolase Activity | 4.16 | 1,053 | 5.21 | 48,643 | 5.27 | 24,634 |
|  | GO:0005215 | Transporter Activity | 2.32 | 587 | 2.84 | 26,545 | 2.85 | 13,321 |
|  | GO:0003677 | DNA Binding | 2.11 | 533 | 2.60 | 24,287 | 2.48 | 11,599 |
|  | GO:0008233 | Peptidase Activity | 1.28 | 324 | 1.54 | 14,349 | 1.78 | 8,307 |

**(B)**

| **Ontology** | **GO-term ID** | **GO-term based Functioinal Annotation** | **Real Sample 4** | | **Real Sample 5** | | **Real Sample 6** | |
| --- | --- | --- | --- | --- | --- | --- | --- | --- |
|  |  |  | Proportion (%) | Count | Proportion (%) | Count | Proportion (%) | Count |
| Biological Process | GO:0008152 | Metabolic Process | 12.93 | 41,184 | 13.60 | 35,745 | 13.45 | 47,108 |
|  | GO:0009058 | Biosynthetic Process | 4.11 | 13,100 | 4.80 | 12,607 | 4.64 | 16,236 |
|  | GO:0006139 | Nucleobase-containing Compound Metabolic Process | 3.62 | 11,536 | 4.46 | 11,732 | 4.44 | 15,533 |
|  | GO:0006810 | Transport | 2.87 | 9,142 | 2.48 | 6,527 | 2.44 | 8,547 |
|  | GO:0006259 | DNA Metabolic Process | 2.40 | 7,631 | 2.68 | 7,048 | 2.76 | 9,678 |
|  | GO:0007165 | Signal Transduction | 2.33 | 7,432 | 1.50 | 3,933 | 1.58 | 5,527 |
|  | GO:0005975 | Carbohydrate Metabolic Process | 2.15 | 6,854 | 1.84 | 4,845 | 1.77 | 6,190 |
|  | GO:0050789 | Regulation of Biological Process | 1.80 | 5,744 | 1.63 | 4,290 | 1.57 | 5,506 |
|  | GO:0009056 | Catabolic Process | 1.72 | 5,482 | 1.86 | 4,888 | 1.82 | 6,371 |
|  | GO:0044238 | Primary Metabolic Process | 1.65 | 5,249 | 2.02 | 5,323 | 1.97 | 6,882 |
| Cellular Component | GO:0005575 | Cellular Component | 5.32 | 16,952 | 4.18 | 11,000 | 4.39 | 15,372 |
|  | GO:0005737 | Cytoplasm | 2.19 | 6,959 | 2.68 | 7,051 | 2.60 | 9,099 |
|  | GO:0005886 | Plasma Membrane | 1.64 | 5,232 | 1.01 | 2,664 | 1.11 | 3,902 |
| Molecular Function | GO:0005488 | Binding | 9.27 | 29,510 | 10.23 | 26,898 | 10.10 | 35,362 |
|  | GO:0003824 | Catalytic Activity | 5.66 | 18,028 | 6.24 | 16,400 | 6.11 | 21,405 |
|  | GO:0016787 | Hydrolase Activity | 5.51 | 17,555 | 5.45 | 14,317 | 5.41 | 18,939 |
|  | GO:0000166 | Nucleotide Binding | 5.32 | 16,932 | 5.96 | 15,663 | 5.88 | 20,590 |
|  | GO:0016740 | Transferase Activity | 4.83 | 15,366 | 5.20 | 13,676 | 5.02 | 17,593 |
|  | GO:0003677 | DNA Binding | 3.19 | 10,155 | 3.22 | 8,476 | 3.25 | 11,388 |
|  | GO:0005215 | Transporter Activity | 3.09 | 9,850 | 2.97 | 7,807 | 2.79 | 9,764 |

**(C)**

| **Ontology** | **GO-term ID** | **GO-term based Functioinal Annotation** | **Simulated Sample 1** | | **Simulated Sample 2** | | **Simulated Sample 3** | |
| --- | --- | --- | --- | --- | --- | --- | --- | --- |
|  |  |  | Proportion (%) | Count | Proportion (%) | Count | Proportion (%) | Count |
| Biological Process | GO:0008152 | Metabolic Process | 14.33 | 26,095 | 13.89 | 38,608 | 14.23 | 28,335 |
|  | GO:0009058 | Biosynthetic Process | 5.45 | 9,929 | 5.15 | 14,306 | 5.37 | 10,685 |
|  | GO:0006139 | Nucleobase-containing Compound Metabolic Process | 4.39 | 7,998 | 4.42 | 12,282 | 4.40 | 8,769 |
|  | GO:0006810 | Transport | 2.62 | 4,770 | 2.70 | 7,512 | 2.63 | 5,229 |
|  | GO:0006259 | DNA metabolic Process | 2.22 | 4,047 | 2.34 | 6,491 | 2.32 | 4,618 |
|  | GO:0044238 | Primary metabolic Process | 1.96 | 3,575 | 1.81 | 5,042 | 1.98 | 3,935 |
|  | GO:0009056 | Catabolic Process | 1.69 | 3,071 | 1.75 | 4,854 | 1.77 | 3,521 |
|  | GO:0019538 | Protein Metabolic Process | 1.34 | 2,433 | 1.34 | 3,723 | 1.29 | 2,561 |
|  | GO:0005975 | Carbohydrate Metabolic Process | 1.29 | 2,350 | 1.39 | 3,850 | 1.27 | 2,524 |
| Cellular Component | GO:0005575 | Cellular Component | 5.03 | 9,161 | 5.04 | 14,019 | 4.92 | 9,797 |
|  | GO:0005737 | Cytoplasm | 2.96 | 5,388 | 2.92 | 8,122 | 2.93 | 5,839 |
| Molecular Function | GO:0005488 | Binding | 10.54 | 19,188 | 10.21 | 28,386 | 10.47 | 20,847 |
|  | GO:0003824 | Catalytic Activity | 6.74 | 12,273 | 6.58 | 18,281 | 6.74 | 13,428 |
|  | GO:0000166 | Nucleotide Binding | 5.79 | 10,553 | 5.71 | 15,880 | 5.77 | 11,481 |
|  | GO:0016740 | Transferase Activity | 5.22 | 9,510 | 5.25 | 14,586 | 5.30 | 10,561 |
|  | GO:0016787 | Hydrolase Activity | 5.03 | 9,157 | 5.27 | 14,635 | 4.99 | 9,944 |
|  | GO:0005215 | Transporter Activity | 2.62 | 4,776 | 2.80 | 7,768 | 2.70 | 5,385 |
|  | GO:0003677 | DNA Binding | 2.31 | 4,202 | 2.43 | 6,755 | 2.40 | 4,771 |
|  | GO:0008233 | Peptidase Activity | 1.48 | 2,697 | 1.68 | 4,677 | 1.38 | 2,749 |

**Table S4. The most abundant SEED based functional annotations of (A) real samples from human saliva environment, (B) real samples from human gut environment and (C) simulated samples**

**(A)**

| **SEED based Functional Annotation** | **Real Sample 1** | | **Real Sample 2** | | **Real Sample 3** | |
| --- | --- | --- | --- | --- | --- | --- |
|  | Proportion (%) | Count | Proportion (%) | Count | Proportion (%) | Count |
| DNA Metabolism | 13.28 | 194 | 20.29 | 5,666 | 19.10 | 4,361 |
| Carbohydrates | 13.48 | 197 | 16.59 | 4,632 | 16.32 | 3,728 |
| Protein Metabolism | 8.56 | 125 | 6.67 | 1,860 | 7.15 | 1,632 |
| Amino Acids and Derivatives | 8.08 | 118 | 5.49 | 1,535 | 4.41 | 1,008 |
| Respiration | 6.02 | 88 | 3.38 | 945 | 3.32 | 758 |
| Iron acquisition and metabolism | 5.20 | 76 | 6.98 | 1,950 | 9.69 | 2,212 |

**(B)**

| **SEED based Functional Annotation** | **Real Sample 4** | | **Real Sample 5** | | **Real Sample 6** | |
| --- | --- | --- | --- | --- | --- | --- |
|  | Proportion (%) | Count | Proportion (%) | Count | Proportion (%) | Count |
| Carbohydrates | 26.33 | 5,172 | 22.19 | 3,351 | 21.91 | 4,708 |
| DNA Metabolism | 12.58 | 2,471 | 15.52 | 2,344 | 15.19 | 3,264 |
| Iron acquisition and metabolism | 11.84 | 2,326 | 2.07 | 313 | 6.08 | 1,307 |
| Amino Acids and Derivatives | 5.77 | 1,134 | 8.21 | 1,239 | 6.60 | 1,419 |
| Protein Metabolism | 5.51 | 1,083 | 6.29 | 949 | 6.77 | 1,455 |

**(C)**

| **SEED based Functional Annotation** | **Simulated Sample 1** | | **Simulated Sample 2** | | **Simulated Sample 3** | |
| --- | --- | --- | --- | --- | --- | --- |
|  | Proportion (%) | Count | Proportion (%) | Count | Proportion (%) | Count |
| DNA Metabolism | 21.47 | 1,860 | 20.31 | 2,853 | 20.44 | 2,070 |
| Carbohydrates | 13.56 | 1,175 | 14.77 | 2,075 | 13.20 | 1,337 |
| Protein Metabolism | 6.75 | 585 | 6.32 | 888 | 6.60 | 669 |
| Amino Acids and Derivatives | 5.50 | 477 | 5.67 | 797 | 6.69 | 678 |
| Nucleosides and Nucleotides | 5.01 | 434 | 5.01 | 704 | 5.15 | 521 |
| RNA Metabolism | 4.71 | 408 | 4.16 | 584 | 4.45 | 451 |
